# Supplementary figures and images for: Pyrazinoic Acid Inhibits the Bifunctional Enzyme (Rv2783) in Mycobacterium tuberculosis by Competing with tmRNA
Source: Pathogens. 2019 Nov 12;8(4):230. doi: 10.3390/pathogens8040230 (PMC6963974; doi:10.3390/pathogens8040230)

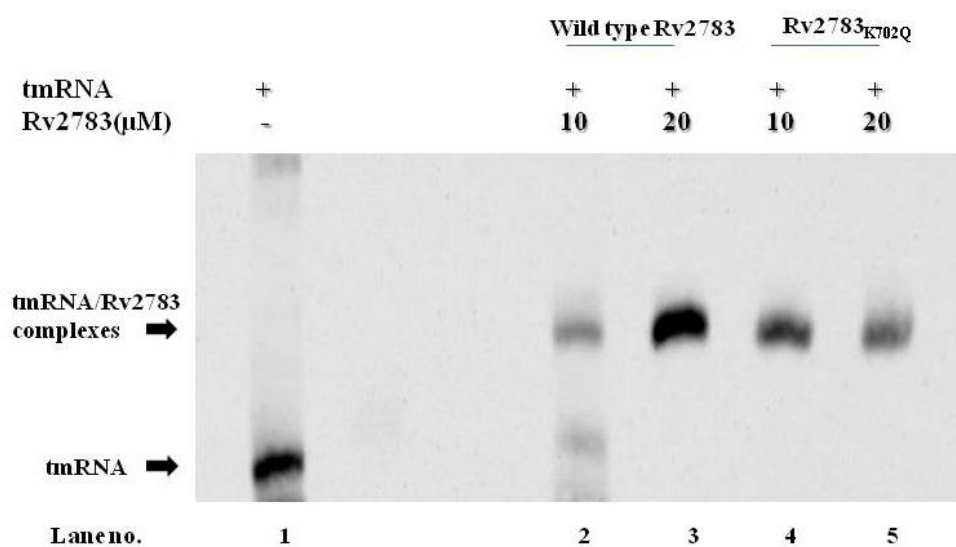

Supplement: Supplementary file 1 [file pathogens-08-00230-s001.pdf]
